# Single-step genome-wide association analyses of claw horn lesions in Holstein cattle using linear and threshold models

**DOI:** 10.1186/s12711-023-00784-4

**Published:** 2023-03-10

**Authors:** Bingjie Li, Matthew Barden, Vanessa Kapsona, Enrique Sánchez-Molano, Alkiviadis Anagnostopoulos, Bethany Eloise Griffiths, Cherril Bedford, Xiaoxia Dai, Mike Coffey, Androniki Psifidi, Georgios Oikonomou, Georgios Banos

**Affiliations:** 1 Department of Animal and Veterinary Sciences, The Roslin Institute Building, Scotland’s Rural College (SRUC), Easter Bush, Midlothian, EH25 9RG UK; 2 Department of Livestock and One Health, Institute of Infection, Veterinary and Ecological Sciences, University of Liverpool, Leahurst Campus, Neston, CH64 7TE UK; 3 The Roslin Institute and R(D)SVS, University of Edinburgh, Easter Bush, Midlothian, EH25 9RG UK; 4 Department of Clinical Science and Services, Royal Veterinary College, Hawkshead Lane, Hatfield, Hertfordshire AL9 7TA UK

## Abstract

**Background:**

Lameness in dairy cattle is primarily caused by foot lesions including the claw horn lesions (CHL) of sole haemorrhage (SH), sole ulcers (SU), and white line disease (WL). This study investigated the genetic architecture of the three CHL based on detailed animal phenotypes of CHL susceptibility and severity. Estimation of genetic parameters and breeding values, single-step genome-wide association analyses, and functional enrichment analyses were performed.

**Results:**

The studied traits were under genetic control with a low to moderate heritability. Heritability estimates of SH and SU susceptibility on the liability scale were 0.29 and 0.35, respectively. Heritability of SH and SU severity were 0.12 and 0.07, respectively. Heritability of WL was relatively lower, indicating stronger environmental influence on the presence and development of WL than the other two CHL. Genetic correlations between SH and SU were high (0.98 for lesion susceptibility and 0.59 for lesion severity), whereas genetic correlations of SH and SU with WL also tended to be positive. Candidate quantitative trait loci (QTL) were identified for all CHL, including some on *Bos taurus* chromosome (BTA) 3 and 18 with potential pleiotropic effects associated with multiple foot lesion traits. A genomic window of 0.65 Mb on BTA3 explained 0.41, 0.50, 0.38, and 0.49% of the genetic variance for SH susceptibility, SH severity, WL susceptibility, and WL severity, respectively. Another window on BTA18 explained 0.66, 0.41, and 0.70% of the genetic variance for SH susceptibility, SU susceptibility, and SU severity, respectively. The candidate genomic regions associated with CHL harbour annotated genes that are linked to immune system function and inflammation responses, lipid metabolism, calcium ion activities, and neuronal excitability.

**Conclusions:**

The studied CHL are complex traits with a polygenic mode of inheritance. Most traits exhibited genetic variation suggesting that animal resistance to CHL can be improved with breeding. The CHL traits were positively correlated, which will facilitate genetic improvement for resistance to CHL as a whole. Candidate genomic regions associated with lesion susceptibility and severity of SH, SU, and WL provide insights into a global profile of the genetic background underlying CHL and inform genetic improvement programmes aiming at enhancing foot health in dairy cattle.

**Supplementary Information:**

The online version contains supplementary material available at 10.1186/s12711-023-00784-4.

## Background

About one in three dairy cows in the United Kingdom (UK) suffer from lameness at any time point [1] and reducing the prevalence of the condition is a key priority for the dairy industry [2, 3]. Lameness is primarily caused by foot lesions [4–6] including sole haemorrhage (SH), sole ulcers (SU), and white line disease (WL) [7–10]. These three lesions, collectively referred to as claw horn lesions (CHL) [11], have been associated with severe pain response [12, 13], reduced milk production [14], compromised reproductive performance [15], and increased risk of premature culling [16] in dairy cattle.

Recent studies in dairy cattle have revealed genetic variation in resistance to CHL, with reported heritability estimates ranging from 0.01 to 0.35 in Holstein cattle [17–19]. Genome-wide association (GWA) analyses have shown a complex genetic background for CHL in dairy cattle [20–24] and possible quantitative trait loci (QTL), but results generally differ between studies, which may be attributed to differences in experimental populations, data sizes and analyses, and trait definition. Previous genetic analyses on CHL mainly used data recorded by farmers or foot-trimmers [17, 20, 21, 24–28]. Although foot-trimming records are undoubtedly a valuable resource for foot lesion management, there are concerns about variability between individual recorders and differences in terminology among studies [29]. Furthermore, mild lesions, which often only require minimal intervention, may be under-recorded when the primary purpose of handling cattle is to perform preventative or therapeutic foot-trimming. In addition, not all cows on a farm may be foot-trimmed during a visit, which may introduce bias in the estimation of genetic variance and genetic parameters for CHL.

In the present study, we closely monitored a large cohort of Holstein dairy cows across multiple lactations and collected detailed foot lesion records from individual animals. The objectives of our study were to (i) estimate genetic parameters for different phenotype definitions of SH, SU, and WL using linear and threshold models, and (ii) characterise the genomic architecture that underlies SH, SU, and WL phenotypes with single-step GWA analyses and post-GWA functional enrichment analyses.

## Methods

### Animals and data recording

In total, 2352 Holstein dairy cows were prospectively enrolled in four herds in the northwest of the UK. The four herds were selected for participation based on practicalities of frequent data collection; animals were enrolled between April and December 2019. A few animals that were enrolled prior to calving but did not subsequently calve due to health issues were excluded from further analyses. Consequently, data on a total of 2305 cows were considered in the present study. The number of animals by herd and lactation number are in Table 1. Some of the herds had been expanding their milking herd before the start of our study, resulting in more second lactation cows being included in our data than first lactation animals. Pedigree information spanning seven generations was extracted from the national database for all studied animals.

**Table 1:** Number of cows by herd and lactation number in the studied population

Lactation number	Number of cows				
	Herd A	Herd B	Herd C	Herd D	Total
1	36	70	445	51	602
2	37	161	462	63	723
3	23	75	237	48	383
4	24	57	191	32	304
5	7	34	86	17	144
6 and more	5	19	108	17	149
Total	132	416	1529	228	2305

Data collection was performed from February 2019 to July 2020. Individual animals were assessed for foot lesions at four time points (one inspection per time point): (1) prior to calving (pre-calving stage, range from 0 to 120 days before calving, mean = 55.3 days before calving); (2) immediately after calving (calving stage, range from 0 to 21 days in milk, mean = 5.4 days in milk); (3) near peak yield (early lactation stage, range from 50 to 120 days in milk, mean = 83.9 days in milk); and (4) latter part of lactation (late lactation stage, range from 170 to 305 days in milk, mean = 199.6 days in milk). A few records that fell outside of the planned sampling time frame were removed from the final data analyses. Consequently, the number of cows included at each stage was 2277 for pre-calving stage, 2185 for calving stage, 2124 for early lactation stage, and 1931 for late lactation stage. The relatively smaller number of phenotyped cows at the late lactation stage was due to temporary movement restrictions for farm visits during the COVID-19 pandemic.

During the visit, animals were restrained in a foot-trimming crush and lesions on each claw were recorded by qualified veterinary surgeons using case definitions described in the International Committee for Animal Recording (ICAR) claw health atlas [30]. All four feet were examined for each lesion and were scored according to lesion severity (Table 2): 0 (absence of lesion), 1 (mild lesion), 2 (moderate lesion), and 3 (severe lesion). Over 90% of foot lesion identification and recording was performed by the same veterinarian. Data collection was the same at all time points with the exception of calving stage on Herd C where only the hind feet were assessed to reduce the handling time of large numbers of newly calved cows on the herd each week.

**Table 2:** Case definitions and severity scoring system for assessing sole haemorrhage (SH), sole ulcers (SU), and white line disease (WL)

Severity grading score	SH (sole haemorrhage)	SU (sole ulcers)	WL (white line disease)
	Discoloration of the sole horn	Exposure of fresh or necrotic corium	Lesion localised to the white line region
0	No lesion	No lesion	No lesion
1	Light pink lesion < 2 cm diameter or diffuse discoloration of sole	< 2 cm diameter lesion covered by a thin layer of horn prior to modelling	Haemorrhage of the white line, discoloration or separation of the white line which disappears after limited trimming
2	Light pink lesion ≥ 2 cm diameter or dark pink/purple lesion < 2 cm diameter	≥ 2 cm diameter lesion with < 1.5 cm granulation tissue protruding through the horn	Deeper separation or discoloration of the white line, lesion is still present after limited trimming
3	Dark pink/purple lesion ≥ 2 cm diameter or discoloration with blue tinge	≥ 1.5 cm granulation tissue protruding through the horn or secondary bacterial infection	Separation of the white line which extends to the corium, purulent exudate or necrotic tissue may be present

### Trait definition

Two phenotypes were defined for each CHL: lesion susceptibility and lesion severity.

#### Lesion susceptibility (binary trait)

Lesion susceptibility classified animals as either affected (case, 1) or unaffected (control, 0) by each CHL over the whole study period. Animals were considered affected if the lesion was present on any foot at any time point, regardless of the lactation stage or the severity of the lesion. Animals were considered unaffected if the lesion was absent on all feet at all four time points. Based on this trait definition, each animal had one lesion susceptibility record (0 or 1) for each CHL. Repeated measurements of the same CHL at multiple time points were not taken into account.

#### Lesion severity (continuous trait)

This phenotype was calculated as the average score of each lesion across all assessed feet of an individual cow. The trait definition accounts for both the number and severity of the lesions, providing extra information on the foot health status of each individual. The phenotype was calculated separately at each time-point of foot examination, so each animal had repeated records of lesion severity at multiple lactation stages.

### Genotypes and quality control

Genome-wide genotypes were available for 2250 animals among all the phenotyped animals. The genotypes had been imputed to 80K single nucleotide polymorphism (SNP) genotypes using the platform for the UK national dairy cattle genomic evaluation. The 80K SNP panel (79,051 SNPs spanning the entire bovine genome), used for national dairy cattle genomic evaluation in the UK, was developed based on Illumina BovineSNP50 BeadChip, 777K Illumina BovineHD BeadChip (Illumina Inc., San Diego, CA), and other commercial genotyping arrays, extra gene tests, and large-effect sequence variants [31]. The chromosome location of the SNPs in the 80K panel uses the latest assembly of the *Bos taurus* genome (ARS-UCD 1.2) [32]. Genotype quality control removed SNPs and animals with call rates lower than 0.90, SNPs with a minor allele frequency lower than 0.05, SNPs with a significant deviation from Hardy–Weinberg equilibrium (*P* < 1E − 6), and animals with parent-progeny Mendelian conflicts. After quality control, 65,211 SNPs for 2167 genotyped individuals were retained for further analyses.

### Estimation of genetic parameters and genomic breeding values

#### Lesion susceptibility

As binary phenotypes, lesion susceptibility for SH, SU, and WL was analysed using a threshold model on a latent liability scale [33]. A Markov chain Monte Carlo approach was used to obtain posterior distributions for model parameters via the Gibbs sampling algorithm in the THRGIBBS1F90 software (version 2.118) [34]. A chain length of 500,000 iterations with a 50,000 sample burn-in produced consistent results, and convergence of Gibbs sampling was assessed using the *coda* package in R (version 4.2) [35]. A thinning interval of 50 samples was used to reduce the lag correlation between consecutive samples. Using the THRGIBBS1F90 software, variance components and heritability for lesion susceptibility of SH, SU, and WL on the liability scale were estimated with univariate analyses, and the animals’ estimated genomic breeding values (GEBV) for each trait were obtained afterwards using the estimated variance components [34]. Genetic correlations between SH, SU, and WL were estimated with bivariate analyses. In all cases, the threshold model used was:

1 $${\boldsymbol{\lambda }} = {\mathbf{Xb}} + {\mathbf{Za}} + {\mathbf{e}},$$

where ${\boldsymbol{\lambda}}$ is a vector of unobserved liabilities for lesion susceptibility of SH, SU, or WL; $\mathbf{b}$ is a vector of the fixed effects of parity and herd-year-season of calving; $\mathbf{a}$ is a vector of the random additive genetic effect with $\mathrm{var}(\mathbf{a})\sim \mathrm{N}(0, \mathbf{H}{\upsigma }_{a}^{2}$), where ${\upsigma }_{a}^{2}$ is the additive genetic variance and $\mathbf{H}$ is the relationship matrix incorporating pedigree and genomic information [36]; $\mathbf{e}$ is a vector of random residuals with $\mathrm{var}(\mathbf{e})\sim \mathrm{N}(0, \mathbf{I}{\upsigma }_{e}^{2})$ where ${\upsigma }_{e}^{2}$ is the residual variance; and $\mathbf{X}$ and $\mathbf{Z}$ are incidence matrices relating records to $\mathbf{b}$ and $\mathbf{a}$, respectively.

#### Lesion severity

Variance components and genetic parameters for lesion severity of SH, SU, and WL (continuous phenotypes) were analysed with linear models using the average information-restricted maximum likelihood (AIREML) algorithm implemented in the AIREMLF90 software (version 1.148) [37]. An animal repeatability model was used to take the repeated lesion measurements for individuals at multiple time-points into account. The animals’ GEBV for each trait were estimated afterwards using the BLUPF90 software (version 1.70) [37] based on the same model with the estimated variance components. The animal repeatability model used was:

2 $$\mathbf{y}=\mathbf{X}\mathbf{b}+\mathbf{Z}\mathbf{a}+\mathbf{W}\mathbf{p}\mathbf{e}+\mathbf{e},$$

where $\mathbf{y}$ is a vector of the lesion severity phenotype of SH, SU, or WL; $\mathbf{b}$ is a vector of fixed effects of parity, lactation stage (four lactation stages) nested within parity, and herd-year-season effect of recording; $\mathbf{a}$ is a vector of the random animal additive genetic effect with $\mathrm{var}(\mathbf{a})\sim \mathrm{N}(0, \mathbf{H}{\upsigma }_{a}^{2})$, where ${\upsigma }_{a}^{2}$ is the additive genetic variance and $\mathbf{H}$ is the relationship matrix incorporating pedigree and genomic information [36]; $\mathbf{p}\mathbf{e}$ is a vector of the random permanent environmental effect with $\mathrm{var}(\mathbf{p}\mathbf{e})\sim \mathrm{N}(0, \mathbf{I}{\upsigma }_{pe}^{2}$), where ${\upsigma }_{pe}^{2}$ is the permanent environmental variance and $\mathbf{I}$ is an identity matrix; $\mathbf{e}$ is a vector of the random residual with $\mathrm{var}(\mathbf{e})\sim \mathrm{N}(0, \mathbf{I}{\upsigma }_{e}^{2})$ where ${\upsigma }_{e}^{2}$ is the residual variance; and $\mathbf{X}$, $\mathbf{Z}$, and $\mathbf{W}$ are incidence matrices relating records to $\mathbf{b}$, $\mathbf{a}$, and $\mathbf{p}\mathbf{e}$, respectively.

### Principal component analyses

**Fig. 1:**
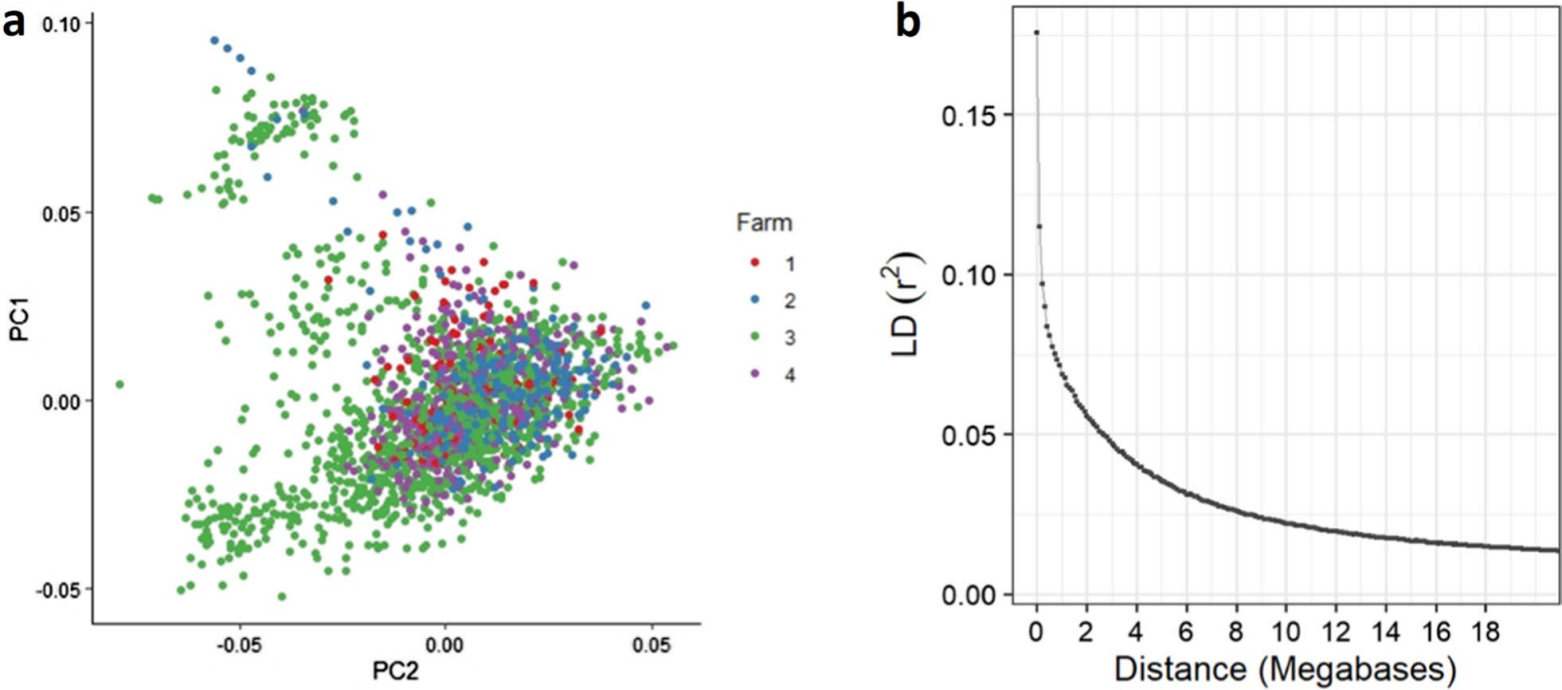
**a** Structure of the studied population explored with principal component analyses (PCA) using the genotypes of all animals. **b** Linkage disequilibrium (LD) decay between markers in the studied population

The structure of the studied population was explored with principal component analyses (PCA) using all animals’ genotypes and the GEMMA software [38]. The principal component analyses revealed no distinct clusters in the studied population (Fig. 1a). Possible groupings of the genotyped animals by farm, parity, age, and sire group were carefully investigated and no distinct clusters attributable to each variable were revealed. The proportion of the variance explained by each principal component (PC) was small with the top five PC explaining 1.62, 1.19, 1.13, 1.03 and 0.94% of the total variance, respectively.

### Single-step genome-wide association analyses

The single-step genome-wide association method (ssGWAS) was applied to estimate single marker effects on each trait by back-solving the animals’ GEBV [39], as implemented with the POSTGSF90 software (version 1.73) [40]. The ssGWAS accounts for population structure and uses all data available by effectively combining the genomic and pedigree relationship matrix among individual animals and information from genotyped and non-genotyped animals with phenotypes [39, 41–44]. The *P*-values of marker effects were calculated for lesion severity of SH, SU, and WL using the POSTGSF90 software (version 1.73) [42]. To account for any potential remaining inflation, the ratio of the median of the empirically observed distribution of the test statistic to the expected median (i.e., inflation factor λ) was used to correct for inflation [45]. A genome-wide significance threshold at *P*-value = 7.67E−07 (*P*-value = 0.05/total number of tested markers, i.e., Bonferroni correction for multiple testing) and a suggestive significance threshold at *P*-value = 1.53E−05 (*P*-value = 1/total number of tested markers, i.e., one false positive per genome scan and Bonferroni correction for multiple testing) were applied. Specifically for the binary lesion susceptibility traits, the *P*-values of marker effects were not applicable to single-step GWA analyses when a threshold model with Gibbs sampling was used. Instead, the standardized SNP effects were calculated using the estimated SNP effects divided by the empirical standard deviation of SNP effects in the POSTGSF90 software (version 1.73) [37].

In addition to assessing single marker effects, 0.65-Mb sliding genomic windows were constructed to calculate the corresponding proportion of genetic variance explained by each window. The 0.65-Mb window size was determined based on the average distance in the genome where linkage disequilibrium (LD) between markers was halved (Fig. 1b), using all studied genotypes evaluated with the PLINK software (version 1.07) [46]. In addition, extra window sizes (e.g., ranging from 0.5 to 1 Mb) were also tested, with the results on top genomic windows remaining the same as those from the 0.65-Mb window. For each lesion trait, the proportion of total genetic variance explained by each sliding window was calculated [41] using the POSTGSF90 software (version 1.73) [40].

### Functional annotation analyses

Candidate genes located in the vicinity of large-effect variants within 0.2 Mb upstream and downstream or within the candidate windows from GWA analyses were identified using the USCS Genome Browser [47] based on the latest *Bos taurus* genome assembly of ARS-UCD1.2 and NCBI *Bos taurus* Annotation Release 106. Gene functional annotation was performed using the UniProt database (Release 2022_04) [48]. The candidate gene list associated with CHL was further examined by performing functional enrichment analyses using the DAVID bioinformatic resource (Release v2022q3) [49, 50] to highlight biological processes and pathways underlying the candidate genes.

## Results

### Lesion prevalence and severity across lactation

The prevalence of SH and WL was generally higher than that of SU in the studied population (Table 3**)**. The highest SH prevalence (57.5%) and most severe SH lesions appeared at the early lactation stage. For SU, lesion prevalence and severity were similar in early and late lactation stages, both higher than those in pre-calving and calving stages. For WL, the prevalence increased from calving (30.3%) to early lactation (37.8%) and then to late lactation (59.2%). The average severity of WL also tended to increase from calving and early lactation stages to the late lactation stage.

**Table 3:** Prevalence (%) and average lesion severity of sole haemorrhage (SH), sole ulcers (SU), and white line disease (WL) at each lactation stage (pre-calving stage, calving stage, early lactation stage, and late lactation stage) in the studied population

Lesion		Pre-calving	Calving	Early	Late	Overall
SH	Prevalence (%)	32.5%	33.0%	57.5%	52.9%	83.0%
	Average lesion severity^a^	0.140	0.189	0.328	0.269	–
SU	Prevalence (%)	3.9%	2.5%	6.1%	6.0%	13.9%
	Average lesion severity	0.015	0.015	0.022	0.027	–
WL	Prevalence (%)	31.0%	30.3%	37.8%	59.2%	88.9%
	Average lesion severity	0.134	0.177	0.163	0.285	–

^a^The lesion severity of an individual was calculated as the average lesion score of all assessed feet of an animal

### Genetic parameters of claw horn lesions

For lesion susceptibility traits, SH and SU were found to be under genetic control with low to moderate heritability estimates (Table 4). These estimates are expressed on the underlying trait liability scale. The genetic correlation between SH and SU susceptibility was high at 0.98 (Table 5). Compared to SH and SU, the genetic variance and heritability for WL susceptibility tended to be lower and were not statistically different from zero (Table 4). The genetic correlation of WL with SU was also positive at 0.70 (posterior standard deviation = 0.20). The genetic correlation of WL with SH was estimated to be 0.20 but with a large posterior standard deviation of 0.27 (Table 5).

**Table 4:** Estimates of additive genetic variance (Va), permanent environmental variance (Vpe), residual variance (Ve), and heritability (h^2^) for lesion susceptibility and severity of sole haemorrhage (SH), sole ulcers (SU), and white line disease (WL)

Trait	Model	Lesion	Va	Vpe	Ve	h^2^
Lesion susceptibility^a^ (binary trait)	Threshold model	SH	0.42 (0.14)	–	1.01 (0.04)	0.29 (0.07)
		SU	0.56 (0.20)	–	1.01 (0.05)	0.35 (0.08)
		WL	0.12 (0.08)	–	1.01 (0.04)	0.10 (0.06)
Lesion severity^b^ (continuous trait)	Linear model	SH	0.012 (0.002)	9.8E−3 (0.002)	0.077 (0.001)	0.12 (0.02)
		SU	0.001 (2E−4)	0.003 (3E−4)	0.008 (1E−4)	0.07 (0.02)
		WL	0.003 (8E−4)	0.003 (0.001)	0.069 (0.001)	0.04 (0.01)

Posterior standard deviations of estimates (for lesion susceptibility traits) or standard errors of estimates (for lesion severity traits) are in brackets

^a^Lesion susceptibility classified animals as either affected at any time point (case, 1) or unaffected throughout the period of study (control, 0); estimates are expressed on the liability scale

^b^The lesion severity of an individual was calculated as the average lesion score of all assessed feet

**Table 5:** Genetic correlations between sole haemorrhage (SH), sole ulcers (SU), and white line disease (WL) for lesion severity (above the diagonal) and lesion susceptibility (below the diagonal)

	SH	SU	WL
SH	–	0.59 (0.17)	0.03 (0.27)
SU	0.98 (0.04)	–	0.67 (0.33)
WL	0.20 (0.27)	0.70 (0.20)	–

Standard errors of estimates (for lesion severity traits) or posterior standard deviations of estimates (for lesion susceptibility traits) are in brackets

For lesion severity, SH, SU, and WL were found to be under genetic control with heritability estimates of 0.12 (SE = 0.02), 0.07 (SE = 0.02), and 0.04 (SE = 0.01), respectively (Table 4). The genetic correlations of the lesion severity of SU with SH and WL were 0.59 and 0.67, respectively (Table 5). The genetic correlation of the lesion severity between SH and WL was not statistically different from zero (Table 5).

### Single-marker genome-wide association results

**Fig. 2:**
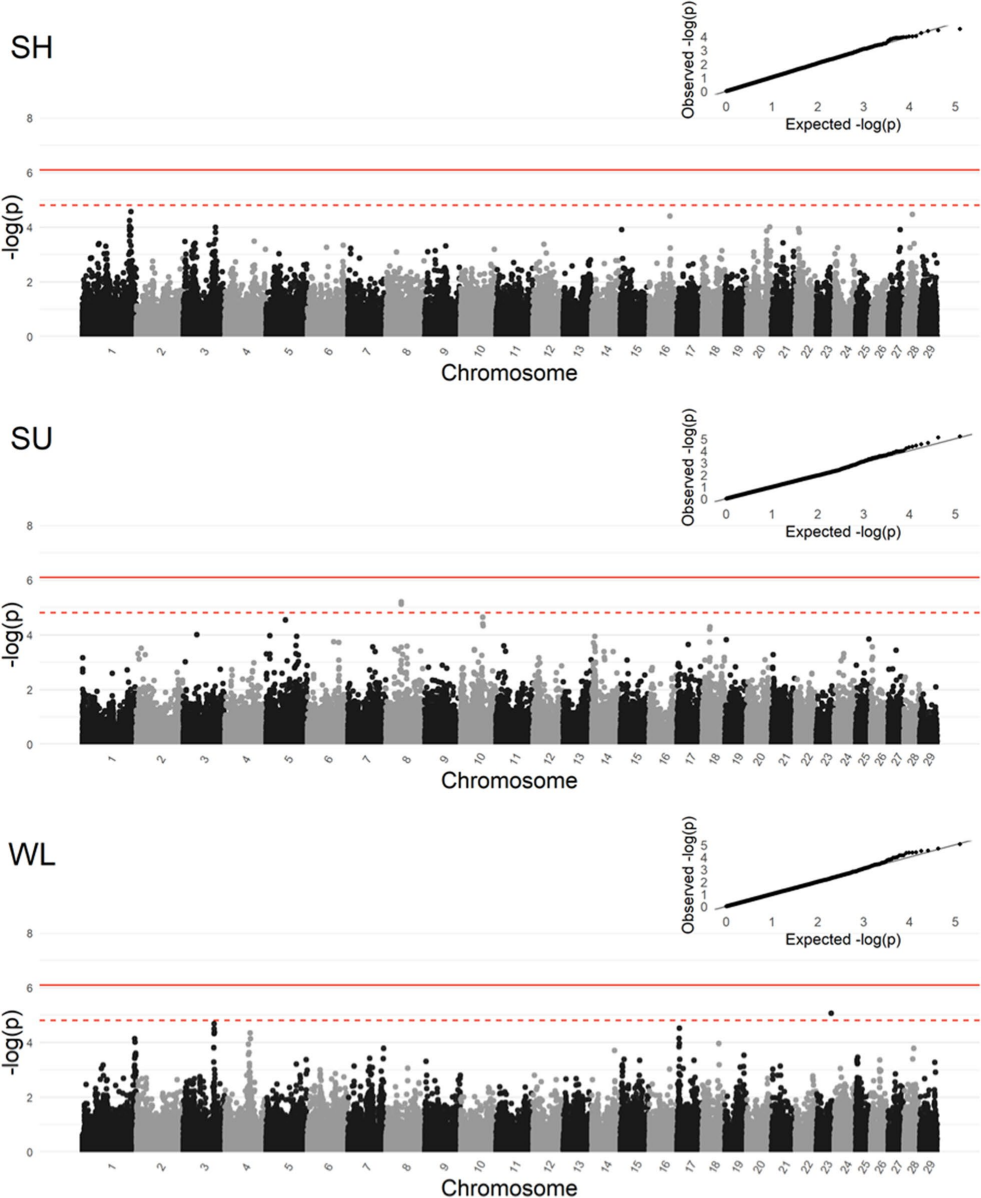
Manhattan plots and quantile–quantile (Q-Q) plots of lesion severity of sole haemorrhage (SH), sole ulcers (SU), and white line disease (WL). The solid line represents the genome-wide significance threshold (P-value = 7.67E−07) and the dashed line represents the suggestive significance line (P-value = 1.53E−05)

Single-marker GWA results for each lesion susceptibility and severity trait are illustrated in Additional file 2: Fig. S1 and Fig. 2, respectively. Furthermore, the top ten markers associated with each lesion severity and susceptibility trait are summarised in Additional file 1: Tables S1 and S2, respectively.

No genome-wide significant markers were identified for the lesion severity traits, although certain peaks of interest reached the suggestive significance threshold (Fig. 2). Two such markers were found associated with SU severity on *Bos taurus* chromosome (BTA) 8 at 44,652,431 bp and 44,735,178 bp, and one marker was found associated with WL severity on BTA23: 43,909,068 bp. However, the signal on BTA23 for WL severity appeared to be a single significant marker without a trailing tail of adjacent markers, and this result needs to be viewed with caution. No single marker was significantly associated with any lesion susceptibility trait (see Additional file 2: Fig. S1).

In addition to the markers reaching a certain level of statistical significance presented above, other large-effect markers of potential interest were observed for SH severity on BTA1: 137,395,018–141,713,562 bp, SU severity on BTA10: 65,020,001–65,180,176 bp and BTA18: 21,483,055–22,535,135 bp, for WL severity on BTA3: 89,579,909–90,937,024 bp and BTA17: 6,204,290–7,269,721 bp, with most of these signals being also observed in the subsequent window-based GWA results. These results may be of interest considering the strict and conservative nature of the Bonferroni correction applied to the significance thresholds in single-marker GWA.

### Window-based genome-wide association results

**Fig. 3:**
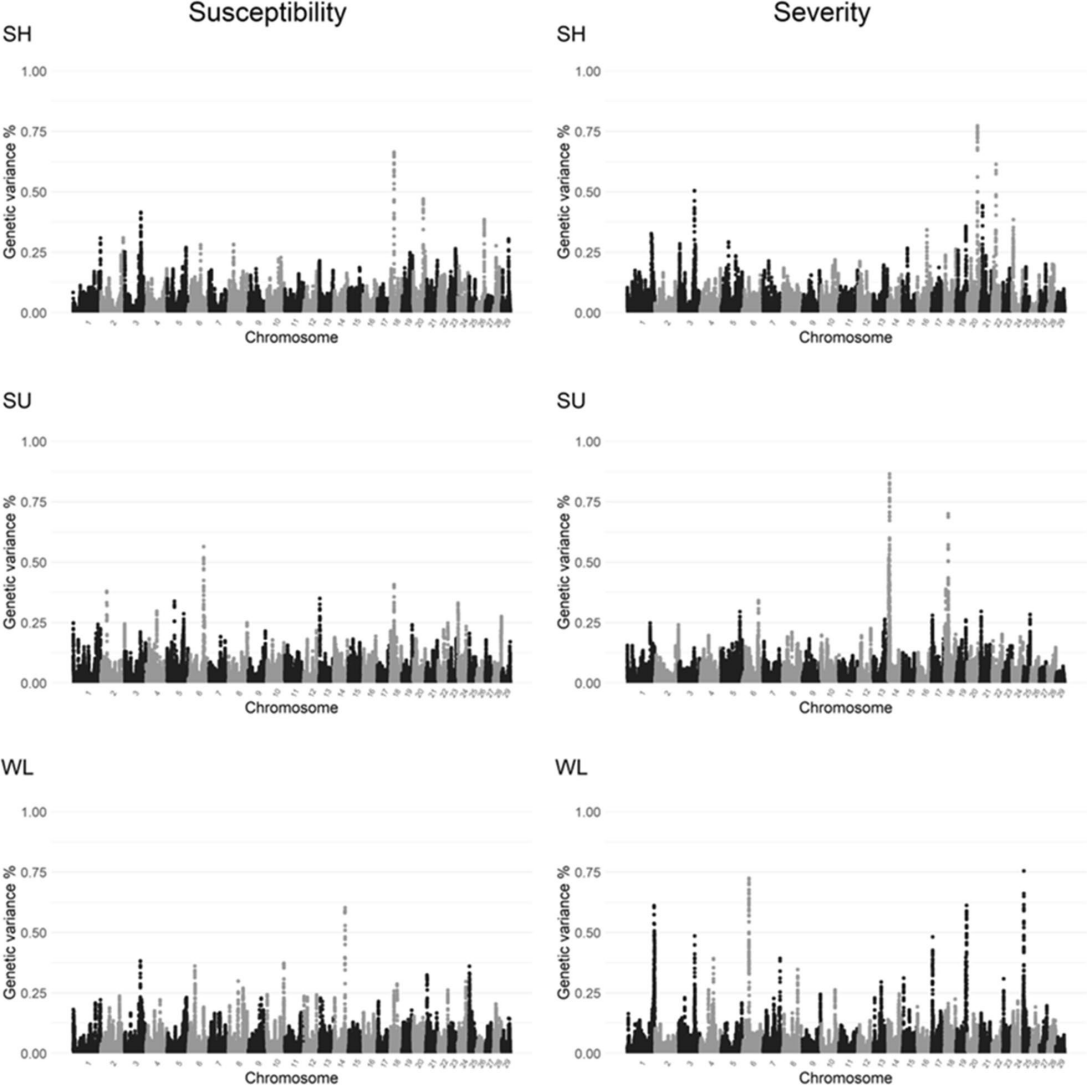
Manhattan plots of the proportion of genetic variance (%) explained by each genomic window of 0.65 Mb for lesion susceptibility (left) and lesion severity (right) of sole haemorrhage (SH), sole ulcers (SU), and white line disease (WL)

Window-based GWA revealed a clearer picture of genomic regions that explained quantifiable proportions of the genetic variance for each lesion (Table 6; Fig. 3). Among the top windows, two genomic regions located on BTA3: 88,750,416–91,356,392 bp and BTA18: 21,454,669–22,223,573 bp (Table 6) were associated with multiple lesions. Specifically, windows within the former region on BTA3 explained a relatively large genetic variance for SH susceptibility and severity, and WL susceptibility and severity. This is consistent with the single-marker GWA results for which large-effect markers (see Additional file 1: Table S1) with clear peaks (Fig. 2) were observed within or flanking the region on BTA3 associated with SH and WL severity. The top genomic window within this region on BTA3 explained 0.41, 0.50, 0.38, and 0.49% of the genetic variance for SH susceptibility, SH severity, WL susceptibility, and WL severity, respectively.

**Table 6:** Genomic windows^a^ that explain the highest proportion of genetic variance (Var%) for sole haemorrhage (SH), sole ulcer (SU), and white line disease (WL), including chromosome (BTA), positions of the window (Pos_start, Pos_end), and *Bos taurus* RefSeq gene(s) within the window

Lesion	Trait	BTA	Pos_start	Pos_end	Var%	RefSeq gene(s) within the window
SH	Lesion susceptibility	18	21,574,466	22,223,573	0.66	*RBL2*, *CHD9*, *AKTIP*, *FTO*
		20	58,161,594	58,795,440	0.47	*ANKH*, *OTULIN*, *OTULINL*, *TRIO*
		3	90,725,628	91,356,392	0.41	*BSND*, *TMEM61*
	Lesion severity	20	58,161,594	58,795,440	0.77	*ANKH*, *OTULIN*, *OTULINL*, *TRIO*
		22	21,284,764	21,911,533	0.61	*ITPR1*, *BHLHE40*, *SUMF1*
		3	88,750,416	89,386,272	0.50	*C8B, C8A*
		21	14,912,081	15,547,515	0.44	*SLCO3A1*
		24	6,860,561	7,499,698	0.38	*RTTN*
		16	53,817,301	54,451,982	0.34	*PDPN*
		1	137,657,855	138,268,870	0.33	*MIR2288*
SU	Lesion susceptibility	6	86,762,457	87,405,290	0.56	*GC, SLC4A4*
		18	21,422,708	22,070,855	0.41	*RBL2, CHD9, AKTIP, FTO*
	Lesion severity	14	8,954,477	9,597,429	0.86	*KCNQ3, EFR3A*
		18	21,454,669	22,081,129	0.70	*RBL2, CHD9, AKTIP, FTO*
		14	5,922,777	6,526,644	0.52	*KHDRBS3*
WL	Lesion susceptibility	14	64,061,208	64,708,627	0.60	*POLR2K*, *RNF19A*
		3	88,750,416	89,386,272	0.38	*C8B, C8A*
		10	97,152,695	97,799,000	0.37	*–*
		6	37,796,921	38,426,291	0.36	*–*
		25	4,353,128	4,984,105	0.30	*–*
	Lesion severity	25	4,231,415	4,872,654	0.75	*–*
		6	37,412,062	38,055,681	0.72	*LCORL*
		19	59,814,966	60,461,025	0.61	*–*
		1	153,405,778	154,054,353	0.61	*PLCL2*, *DAZL*
		1	155,049,738	155,696,507	0.49	*SATB1*
		3	89,466,489	90,090,412	0.49	*PLPP3*, *PRKAA2*
		17	5,843,870	6,493,219	0.48	*GATB*

^a^Only the top window of each peak was reported. Sliding windows that fall into the same peak due to LD with the top window were not reported

The other genomic region on BTA18: 21,422,708–22,223,573 bp was associated with SH susceptibility, and SU susceptibility and severity. The top genomic window within this region on BTA18 explained 0.66, 0.41, and 0.70% of the genetic variance for SH susceptibility, SU susceptibility, and SU severity, respectively. The same region on BTA18: 21.4–22.5 Mb harbours large-effect markers associated with SU severity in the single-marker GWA results (see Additional file 1: Table S1).

In addition to regions on BTA3 and 18 that were associated with multiple lesions, the genomic window on BTA20: 58,161,594–58,795,440 bp was linked to both SH susceptibility and severity (Table 6). This genomic window explained 0.47 and 0.77% of the total genetic variance for SH susceptibility and SH severity, respectively. For WL, apart from BTA3, regions on BTA6: 37.4–38.4 Mb and BTA25: 4.2–4.9 Mb were related to both WL susceptibility and severity.

### Gene functional annotation and enrichment

Genes located within the top genomic windows or 0.2-Mb upstream/downstream of large-effect variants belong mainly to the group of immune system functions in *Bos taurus* including inflammation response and innate immune response (*GC* and *OTULIN*), complement activation (*C8B* and *C8A*), B cell activities (*PLCL2*), leukocyte migration (*TRIO*) [51], and production of pro-inflammatory cytokines (*PLPP3*). In addition, genes within the top windows are also related to calcium ion activities (*ANKH*, *PRKAA2* and *ITPR1*), neuronal excitability *(KCNQ3*), gene expression (*KHDRBS3*, *POLR2K*, *SATB1* and *RBL2*) and protein biosynthesis (*GATB*).

In the functional enrichment analyses, the identified genes from GWA results are enriched in immune responses of complement activation (GO:0006957; GO:0006958) and cytolysis (GO:0019835) from biological processes of Gene Ontology (GO) terms, and are also enriched in metabolism of lipids (*Bos taurus*) (R-BTA-556833) from the Reactome pathways.

## Discussion

### Genetic parameters for lesion susceptibility and severity

Two types of claw horn lesion phenotypes, one associated with susceptibility and the other with severity, were studied for each of the SH, SU, and WL conditions. Lesion severity phenotypes considered the average lesion severity of all feet at multiple time points before and across lactation since the lesion status of animals was recorded in detail for each foot over time in the studied population. Compared to lesion severity, the lesion susceptibility phenotype focused only on the presence of a lesion (i.e., as 0/1 for healthy/affected) at any time point and on any foot, regardless of lesion severity, number of feet affected, or lactation stage when the animal was affected. The lesion susceptibility phenotype is relatively close to the trait definition derived from foot-trimming records that is commonly used for genetic studies on foot lesions, except that in our study we carefully recorded mild lesions of SH, SU or WL, which may not always be represented in foot-trimming records. We recognize that it would also be possible to use binary lesion records at four time points as repeated measurements to analyse lesion susceptibility with an animal repeatability model, although this approach would increase the chance of mis-phenotyping because less information is used to classify unaffected (healthy) animals. Given this, we explored this approach and found similar heritability estimates for lesion susceptibility considering standard deviation of estimates. Thus, the results of analysing lesion susceptibility using repeated records are not reported in this paper.

Genetic parameters were estimated for lesion susceptibility and lesion severity, respectively. Although heritability estimates for lesion severity traits are not directly comparable to those for lesion susceptibility, since the latter are expressed on an underlying liability scale, both suggest the presence of significant genetic variance among animals, at least for SH and SU, to underpin genetic selection for improved foot health.

Lesion susceptibility for SH and SU was found to be under genetic control with low to moderate heritabilities, in agreement with previous studies on foot lesions in dairy cattle [17, 18, 26–28, 52, 53]. The high positive genetic correlation between SH and SU susceptibility (0.98**)** is in broad agreement with previous studies that reported positive genetic correlations between SH and SU ranging from 0.38 to 0.90 [17, 18, 28, 52–55]. This is also consistent with SU developing from evolved severe cases of SH. For WL susceptibility, the heritability was estimated to be 0.10 in this study but not statistically different from zero. Previous studies reported heritability estimates for WL susceptibility ranging from 0.01 to 0.11 [18], with the variation of these heritability estimates being partly due to differences in the populations studied, limited data sizes, phenotype definitions or phenotyping accuracy. In our study, the susceptibility of WL was shown to be positively genetically correlated with SH and SU, which is consistent with previous studies reporting genetic correlation estimates of 0.06 to 0.73 between SH and WL, and 0.31 to 0.98 between SU and WL [18]. The positive genetic correlations between SH, SU and WL indicated the possibility of genetic improvement of resistance to one lesion through selection on the other.

The variance components and heritability for binary lesion susceptibility traits were estimated on the liability scale using a threshold model to consider the genetic variation underlying the observed case–control phenotype of disease traits. An alternative method is to estimate heritability on the observed scale (i.e., simply 0 and 1) for binary lesion traits and then transform to the liability scale [56]. We tested both methods for estimating the heritability for binary lesion traits and the results agreed with each other.

For lesion severity traits, the lesion severity of SH, SU, and WL was also shown to be under genetic control with low heritabilities. The severity of claw horn lesions has been less studied in previous research mainly due to the nature of foot-trimming records. Similar to lesion susceptibility, the severity of SH and SU was also shown to be positively correlated to each other. The genetic correlation between WL severity and SU severity tended to be positive, inferring genetic association between the severity of the two lesions. In contrast, the genetic correlation between the severity of SH and WL was close to 0 but with uncertainty due to the large standard error, which may be partly affected by the low heritability of WL. Based on previous research, the genetic background of WL-related phenotypes is generally poorly understood with a heritability of WL-related phenotypes ranging from 0.01 to 0.11 with relatively large standard errors [18], which indicates a strong influence of the environment on the presence and development of WL or a lack of accuracy in defining WL phenotypes.

### Genetic architecture of CHL

All GWA results indicated complex genetic architecture underlying lesion susceptibility and severity of SH, SU, and WL, which is consistent with findings from previous studies [19, 21–24]. Nevertheless, although the development of CHL appears to be a multi-factorial process that is potentially controlled by a large number of genetic variants, certain genomic regions were identified in our study that explained relatively large proportions of the genetic variance for the CHL traits. These candidate genomic regions harbour annotated genes that closely link to the immune system function and inflammation response (including production of pro-inflammatory cytokines, activities of neutrophils, complement activities, B cell activities and leukocyte migration), lipid metabolism, calcium ion activities, and neuronal excitability. The role of inflammatory responses in the development of CHL has been previously postulated [57]; a recent study showed a significant association between early lactation clinical mastitis and the subsequent development of SU [58]. The role of lipid metabolism in the development of CHL has also been reported [59, 60]. The link of neuronal excitability with CHL may be associated with animals’ response to pain caused by foot lesions.

Among all the top genomic windows associated with CHL in our study, two regions located on BTA3: 88.7–91.4 Mb and BTA18: 21.4–22.3 Mb may be of particular interest as they were found to be associated with multiple foot lesions. The region on BTA3: 88.7–91.4 Mb that is associated with both SH and WL in our study overlaps with a reported candidate QTL for digital dermatitis, an infectious foot lesion, in Holstein cattle [19]. The region on BTA18: 21.4–22.3 Mb that is associated with both SH and SU in our study was previously found to be related to infectious foot lesions of heel horn erosion in Holstein cattle [22]. These findings might suggest the existence of potential genetic links between the non-infectious foot lesions studied here (SH, SU, WL) and infectious foot lesions such as digital dermatitis and heel horn erosion in dairy cattle. Annotated genes of interest within these two regions are responsible for the production of pro-inflammatory cytokines (*PLPP3* gene) and complement activities (*C8B* and *C8A* genes) in the immune responses of *Bos taurus*. The *PLPP3* gene has also been reported to be associated with resistance to clinical mastitis in Holstein cattle [61].

Certain additional genomic regions identified for CHL in our study were also previously reported. The genomic window on BTA6: 86,762,457–87,405,290 bp that is associated with SU susceptibility overlaps with a candidate QTL previously reported for WL in Holstein cattle [22]. The *GC* gene (*GC vitamin D binding protein*), within this region on BTA6, is linked with the enhancement of neutrophil activities in inflammation and macrophage activation. The *GC* gene on BTA6 has been widely reported to be associated with clinical mastitis in dairy cattle based on large-scale genetic studies in Nordic Holsteins [62] and functional genomic analyses on mastitis resistance in Dutch Holstein Friesians [63]. Results from these and our studies might collectively infer pleiotropic effects of the *GC* gene on the genetic control of multiple conditions.

In addition, the large-effect markers with a clear peak around BTA1:137.3–141.7 Mb that are identified here for SH severity are in high LD with a reported candidate QTL for SH around BTA1: 136.9 Mb that was reported in the French Holstein population [22]. The precise location of candidate QTL needs further fine-mapping of the region using higher-density genotypes including whole-genome sequencing data.

### Insights, limitations, and future perspectives

Genetic parameters, large-effect markers, candidate genomic regions and annotated genes identified for CHL susceptibility and severity phenotypes offer a holistic view of the genetic background of CHL. Based on these results, we found that some of the candidate QTL identified here were shared between susceptibility and severity for a lesion, but mostly they were specific to either its susceptibility or severity. The results might imply potential differences between the genetic basis of lesion susceptibility and lesion severity even if the phenotypes are related to each other. Apart from lesion susceptibility and severity, the phenotype of “lesion recovery”, i.e. the animals’ ability to recover from a foot lesion, was previously studied in Holstein cattle [64]. Results from that study suggested differences in the genetic background between disease recovery and susceptibility [64].

Claw horn lesions in dairy cattle are complex traits with a low heritability that are potentially controlled by multiple genes [18]. We applied window-based GWA to account for the imperfect LD between single SNPs and QTL that affect complex traits [65]. The size of the genomic windows (i.e., 0.65 Mb in this study) was determined based on the LD structure of the studied population. We also tested additional window sizes ranging from 0.5 to 1 Mb, which resulted in the same top genomic windows. However, due to the limitations of the genotype DNA array density, the identified candidate genomic regions were quite large, leading to a certain level of uncertainty regarding the exact location of the QTL and the annotated candidate genes. Future fine-mapping analyses using sequence data are needed to further validate the results and narrow down the candidate regions of interest; for example, for the 500-kb upstream and downstream regions from large-effect SNPs and the top genomic windows reported here. Potential large-effect functional variants may also be further validated using functional genomic analyses, such as gene expression analyses and experimental validation.

Apart from the insights into the genetic basis of CHL, large-effect markers and candidate genomic regions identified for CHL may also inform the development of accurate genomic selection (e.g., offer weights to the markers) towards reducing the incidence of CHL and lameness in dairy cattle populations. Improving functional annotation of the cattle genome and inclusion of biological priors such as variants with functional significance will contribute to improved accuracy of genomic predictions for complex traits in dairy cattle breeding [66]. Precise locations of the QTL associated with CHL will offer insights into the genetic improvement of foot health in dairy cattle and drive further refinements in the design of genotyping chips for selective breeding purposes.

## Conclusions

The studied claw horn lesions, which are responsible for lameness in dairy cattle, are complex traits that follow a polygenic mode of inheritance. Most traits exhibited genetic variation, which suggests that animal resistance to CHL can be improved with selective breeding. The individual CHL traits were generally positively correlated, indicating the possibility to expedite genetic improvement to CHL resistance as a whole. Candidate genes and QTL associated with CHL are linked to the immune system function and inflammation responses, lipid metabolism, calcium ion activities, and neuronal excitability. The presence of some common QTL that are shared between multiple lesions and between lesion susceptibility and severity further corroborates the genetic associations among foot lesions. Candidate QTL identified for lesion susceptibility and severity provide insights into a global picture of the genetic background that underlies CHL and inform genomic selection for improved foot health in dairy cattle.

## Supplementary Information

**Additional file 1: Table S1.** The top ten markers associated with lesion severity for sole haemorrhage (SH), sole ulcer (SU), and white line disease (WL) from single-marker GWA analyses, including chromosome (BTA), position (Pos), minor allele frequency (MAF), and *P*-value**.** The data provided represent the position, allele frequency, and the *P* value of the top ten markers associated with lesion severity for sole haemorrhage (SH), sole ulcer (SU), and white line disease (WL) from the genome-wide association analysis. **Table S2.** The top ten markers with the largest standardized SNP effects (Std_SNP_effect) on lesion susceptibility for sole haemorrhage (SH), sole ulcer (SU), and white line disease (WL) from single-marker GWA analyses, including chromosome (BTA), position (Pos), and minor allele frequency**.** The data provided represent the position, allele frequency, and the effect size of the top ten markers associated with lesion susceptibility for sole haemorrhage (SH), sole ulcer (SU), and white line disease (WL) from the genome-wide association analysis.

**Additional file 2: Figure S1**. Manhattan plots of lesion susceptibility for sole haemorrhage (SH), sole ulcers (SU), and white line disease (WL). The standardized SNP effects were calculated by the estimated SNP effects divided by their empirical standard deviation. The data provided represent the Manhattan plots of the standardized SNP effects for lesion susceptibility of sole haemorrhage (SH), sole ulcers (SU), and white line disease (WL) from the genome-wide association analysis.

## Data Availability

The datasets used and/or analyzed during the current study are available from the corresponding author on reasonable request. The imputed genotype data that support the findings of this study are stored at Scotland’s Rural College (SRUC), but restrictions apply to the availability of the data, which were used under agreement for the current study, and so are not publicly available.
