# Supplementary material for: Single-step genome-wide association analyses of claw horn lesions in Holstein cattle using linear and threshold models
Source: Genet Sel Evol. 2023 Mar 10;55:16. doi: 10.1186/s12711-023-00784-4 (PMC9999328; doi:10.1186/s12711-023-00784-4)
Supplement: Supplementary file 1 — Additional file 1: Table S1. The top ten markers associated with lesion severity for sole haemorrhage (SH), sole ulcer (SU), and white line disease (WL) from single-marker GWA analyses, including chromosome (BTA), position (Pos), minor allele frequency (MAF), and P-value. The data provided represent the position, allele frequency, and the P value of the top ten markers associated with lesion severity for sole haemorrhage (SH), sole ulcer (SU), and white line disease (WL) from the genome-wide association analysis. Table S2. The top ten markers with the largest standardized SNP effects (Std_SNP_effect) on lesion susceptibility for sole haemorrhage (SH), sole ulcer (SU), and white line disease (WL) from single-marker GWA analyses, including chromosome (BTA), position (Pos), and minor allele frequency. The data provided represent the position, allele frequency, and the effect size of the top ten markers associated with lesion susceptibility for sole haemorrhage (SH), sole ulcer (SU), and white line disease (WL) from the genome-wide association analysis. [file 12711_2023_784_MOESM1_ESM.docx]

**Additional file 1**

**Additional file 1 Table S1**

The top ten markers associated with lesion severity for sole haemorrhage (SH), sole ulcer (SU), and white line disease (WL) from single-marker GWA analyses, including chromosome (BTA), position (Pos), minor allele frequency (MAF), marker effect size (Effect), and *P*-value.

| **Lesion** | **BTA** | **Pos (bp)** | **MAF** | ***P*-value^a^** |
| --- | --- | --- | --- | --- |
| SH severity | 1 | 141,365,527 | 0.21 | 2.73E-05 |
|  | 28 | 24,747,891 | 0.42 | 3.45E-05 |
|  | 16 | 60,525,984 | 0.19 | 3.93E-05 |
|  | 1 | 137,395,018 | 0.26 | 5.62E-05 |
|  | 1 | 138,024,984 | 0.41 | 9.23E-05 |
|  | 20 | 67,471,544 | 0.13 | 9.85E-05 |
|  | 3 | 93,668,143 | 0.41 | 1.02E-04 |
|  | 1 | 141,713,562 | 0.34 | 1.10E-04 |
|  | 22 | 9,683,464 | 0.25 | 1.13E-04 |
|  | 1 | 138,056,926 | 0.40 | 1.20E-04 |
| SU severity | 8 | 44,652,431 | 0.08 | 6.31E-06* |
|  | 8 | 44,735,178 | 0.08 | 7.72E-06* |
|  | 10 | 65,020,001 | 0.11 | 2.25E-05 |
|  | 5 | 56,420,099 | 0.06 | 2.86E-05 |
|  | 10 | 65,055,281 | 0.12 | 3.90E-05 |
|  | 10 | 65,180,176 | 0.11 | 4.61E-05 |
|  | 18 | 22,535,135 | 0.23 | 5.08E-05 |
|  | 18 | 21,483,055 | 0.35 | 6.27E-05 |
|  | 3 | 39,904,161 | 0.08 | 9.87E-05 |
|  | 5 | 10,960,727 | 0.12 | 1.08E-04 |
| WL severity | 23 | 43,909,068 | 0.13 | 8.73E-06* |
|  | 3 | 89,815,776 | 0.22 | 2.10E-05 |
|  | 17 | 7,269,721 | 0.35 | 3.07E-05 |
|  | 3 | 90,090,412 | 0.18 | 3.17E-05 |
|  | 3 | 90,937,024 | 0.25 | 4.24E-05 |
|  | 4 | 73,971,874 | 0.20 | 4.48E-05 |
|  | 3 | 89,688,733 | 0.30 | 4.52E-05 |
|  | 3 | 89,579,909 | 0.27 | 4.78E-05 |
|  | 17 | 6,204,290 | 0.32 | 7.16E-05 |
|  | 1 | 152,437,124 | 0.32 | 7.35E-05 |

^a^Genome-wide significance threshold at *P*-value = 7.67E-07 and suggestive significance threshold at *P*-value = 1.53E-05. Markers reaching significance thresholds were marked with asterisk (*).

**Additional file 1 Table S2**

The top ten markers with the largest standardized SNP effects (Std_SNP_effect) on lesion susceptibility for sole haemorrhage (SH), sole ulcer (SU), and white line disease (WL) from single-marker GWA analyses, including chromosome (BTA), position (Pos), and minor allele frequency (MAF)

| **Lesion** | **BTA** | **Pos (bp)** | **MAF** | **Std_SNP_effect^a^** |
| --- | --- | --- | --- | --- |
| SH susceptibility | 7 | 1,796,400 | 0.28 | 4.09 |
|  | 4 | 28,253,982 | 0.35 | 4.08 |
|  | 7 | 12,965,356 | 0.48 | 4.07 |
|  | 4 | 27,890,577 | 0.40 | 4.01 |
|  | 29 | 44,577,341 | 0.40 | 4.00 |
|  | 15 | 68,358,382 | 0.34 | 3.99 |
|  | 5 | 33,560,114 | 0.48 | 3.98 |
|  | 17 | 19,924,694 | 0.49 | 3.95 |
|  | 13 | 79,875,689 | 0.40 | 3.92 |
|  | 7 | 12,994,583 | 0.46 | 3.85 |
| SU susceptibility | 27 | 24,424,298 | 0.45 | 4.47 |
|  | 5 | 4,889,808 | 0.33 | 4.25 |
|  | 5 | 89,051,206 | 0.41 | 4.23 |
|  | 7 | 76,625,461 | 0.38 | 4.21 |
|  | 26 | 31,562,060 | 0.36 | 4.09 |
|  | 5 | 90,375,279 | 0.40 | 4.05 |
|  | 14 | 72,785,677 | 0.45 | 4.05 |
|  | 5 | 90,364,435 | 0.29 | 4.03 |
|  | 8 | 1,782,066 | 0.49 | 4.02 |
|  | 5 | 87,262,266 | 0.36 | 4.02 |
| WL susceptibility | 24 | 12,855,197 | 0.36 | 4.50 |
|  | 18 | 39,385,561 | 0.41 | 4.11 |
|  | 3 | 103,194,072 | 0.44 | 4.09 |
|  | 18 | 39,488,321 | 0.41 | 4.08 |
|  | 18 | 39,440,689 | 0.41 | 4.05 |
|  | 14 | 52,361,610 | 0.48 | 3.97 |
|  | 2 | 99,164,443 | 0.48 | 3.95 |
|  | 21 | 13,283,849 | 0.33 | 3.92 |
|  | 18 | 40,219,814 | 0.21 | 3.91 |
|  | 8 | 88,091,837 | 0.49 | 3.91 |

^a^The standardized SNP effects were calculated by the estimated SNP effects divided by their empirical standard deviation. The *P*-values of SNP effects were not applicable to single-step GWA analyses when a threshold model with Gibbs Sampling was used.
