# Supplementary figures and images for: Single-step genome-wide association analyses of claw horn lesions in Holstein cattle using linear and threshold models
Source: Genet Sel Evol. 2023 Mar 10;55:16. doi: 10.1186/s12711-023-00784-4 (PMC9999328; doi:10.1186/s12711-023-00784-4)

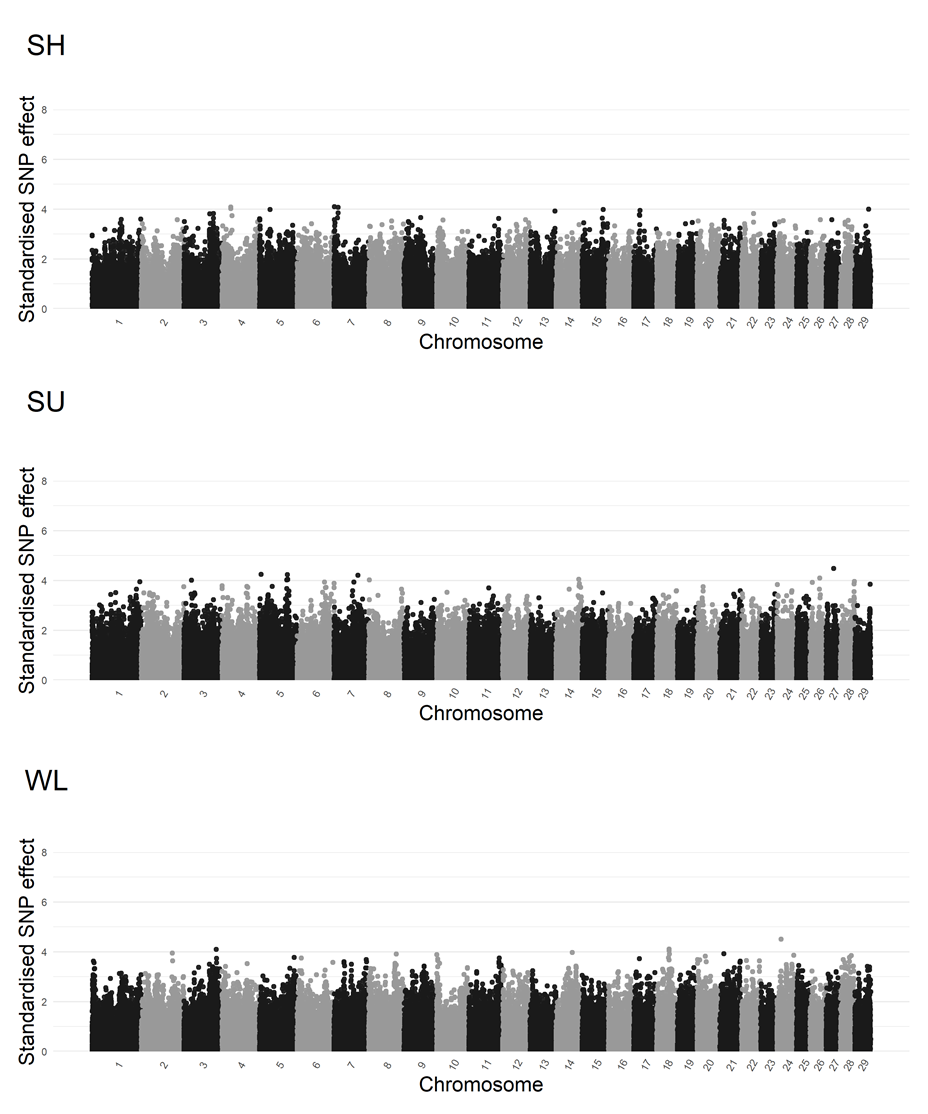

Supplement: Supplementary file 2 — Additional file 2: Figure S1. Manhattan plots of lesion susceptibility for sole haemorrhage (SH), sole ulcers (SU), and white line disease (WL). The standardized SNP effects were calculated by the estimated SNP effects divided by their empirical standard deviation. The data provided represent the Manhattan plots of the standardized SNP effects for lesion susceptibility of sole haemorrhage (SH), sole ulcers (SU), and white line disease (WL) from the genome-wide association analysis. [file 12711_2023_784_MOESM2_ESM.docx]
